# Oral Cancer Awareness Among Dental Patients in Omdurman, Sudan: a cross-sectional Study

**DOI:** 10.1186/s12903-017-0351-z

**Published:** 2017-03-23

**Authors:** Tasneem Mohammed Babiker, Khansa Awad Alkareem Osman, Safa Abdelrawf Mohamed, Matab Abdalrhaman Mohamed, Hatim Mohammed Almahdi

**Affiliations:** Faculty of Dentistry, University of Science and Technology, Omdurman, Sudan

**Keywords:** Oral cancer, Risk factors, Signs and symptoms, Knowledge, Attitude, Oral cancer screening, Smokeless tobacco, Toombak

## Abstract

**Background:**

Oral cancer is a preventable disease. Its occurrence is mostly due to lifestyle. In Sudan, the use of smokeless tobacco (Toombak) has long been linked to oral cancer. Knowledge of the signs and symptoms of oral cancer may well aid in early diagnosis and treatment. This is bound to result in increasing survival rates, as well as reducing the oral cancer burden on the society. This study aimed to assess oral cancer awareness regarding knowledge of signs, symptoms, risk factors and sources of the information. Furthermore, it attempts to evaluate attitudes towards oral cancer screening and any previous experience of screening, amongst dental patients attending University of Science and Technology (UST) Dental Teaching Hospital. Omdurman, Sudan.

**Methods:**

A hospital based cross-sectional study, interviewer-administered questionnaire was conducted amongst 500 adult patients attending the UST Dental Hospital during 2015.

**Results:**

A total of 57.7% (286) of the individuals demonstrated good knowledge of signs and symptoms, whereas 49% (139) expressed good knowledge of risk factors of oral cancer. For the majority of the individuals 66.1% (290), the most common source of information about oral cancer was from the media, while 33.9% individuals (149), obtained knowledge from direct contact of health workers. The overwhelming majority, 93.2% (466) never screened for oral cancer despite their positive attitude towards it 66.4% (332). Knowledge of risk factors associated significantly with those reported positive attitude towards oral cancer screening and those reported direct contact with health workers as a source of information, (*p* ≤ 0.001). Moreover, females and those living in urban districts scores higher than their counterpart in knowledge of risk factor of oral cancer. In addition, those employed 58.6% (280) and 62.8% (164) with correct believes about oral cancer showed significant association with positive knowledge of signs and symptoms (*p* ≤ 0.05).

**Conclusions:**

Awareness levels, knowledge of risk factors and identifying early signs and symptoms of oral cancer necessitate the need for more structured preventive programs using media. Dentists and health workers should do more because they have a pivotal role in early diagnosis by performing oral cancer screening, raising levels of knowledge and in rectifying misconceptions about oral cancer. This would entail a reduction in high rates of morbidity and mortality associated with oral cancer.

**Electronic supplementary material:**

The online version of this article (doi:10.1186/s12903-017-0351-z) contains supplementary material, which is available to authorized users.

## Background

Oral cancer (OC) which includes cancers of the lip, tongue and rest of the oral cavity, but not cancers of the major salivary glands [1], is responsible for sizeable morbidity and mortality rates worldwide especially in developing countries. While it is estimated that cancer incidence 14 million new cases, oral cancer alone claims about 300.000 deaths (2.1%) annually with 1.8% mortality worldwide [2, 3].

Oral cancer in Sudan is ranked as the sixth amongst all cancers types (6.1 per 100.000) [4]. This is strongly attributed to the use of local type of smokeless tobacco (SLT) known as Toombak, which is popular in the Sudanese community. Toombak is made from finely ground leaves of *Nicotiana rustica*, and is mixed with natron or atron (sodium bicarbonate) and water. *Natron* or atron is probably added to Toombak for its alkaline effect and for fast absorption of nicotine to the central nervous system. Tobacco-specific nitrosamines (TSNA) levels in Sudanese Toombak were found to be unusually high compared to the reported levels in any other SLT. The etiologic association between Toombak use and oral cancer has been investigated by several studies [5–8].

Most of the oral cancer cases and deaths due to the individual susceptibility, linked to specific genetic attributes and exposure to carcinogens brought about by lifestyle behaviors [9].

Lifestyle behavior risk factors associated with oral cancer and other determinants of the disease, are interrelated with public knowledge of this disease [10]. Age, gender, tobacco use (smoked and smokeless), alcohol, infection (HPV, candida), lower socio-economic status, unhealthy diet with low fruit and vegetable intake, lack of physical activity are among the known risk factors for oral cancer [11, 12].

The oral cavity is easily accessible for self or clinical examination to detect lesions that are potentially malignant which can make early detection and diagnosis of the oral cancer achievable. Subsequently, this can significantly reduce the diagnostic delays of oral cancer which estimated to be 50% of cases [13–15]. Screening for oral cancer by visual and palpation assessment is still controversial as there is no evidence of the effectiveness of such assessment in reducing mortality from oral cancer. However, it is still recommended that dentists should “remain vigilant for signs of potentially malignant disorders (PMD) and oral cancer while performing routine oral examinations in practice” [1, 16, 17].

Typical signs and symptoms of oral cancer includes, white and red patches on the lining of the oral mucosa, unhealed oral ulcers, swellings of the mouth, loosening of one or more teeth without obvious reason, jaw pain and stiffness, difficulty or pain in swallowing, speech difficulties, reduced mobility of the tongue, numbness of the tongue or teeth or lips, bleeding of unknown origin, neck swelling, fetor oris, altered dental occlusion, sore throat, painful tongue, hoarse voice and persistent neck pain [12].

Oral cancer is a preventable disease along with increased knowledge of oral cancer risk factors, signs and symptoms and this in turn is directly related to the prognosis of the cases identified. This is due to the fact that reinforcement of awareness on oral cancer can possibly lead to detection of early clinical presentation and hence early diagnosis. Moreover, oral cancer can be reduced by limiting the risk factors and early detection of signs and symptoms [1, 18].

This study was conducted in Omdurman, which is the largest city in Khartoum state, the capital of Sudan. It consists of three administrative localities; Omdurman, Umbadda and Karary. A total of more than 2 million inhabitants (2.215.33) account for almost 42% of population of the capital [19]. The University of Science and Technology (UST) Dental Teaching Hospital is serving around 15.000 patients a year.

Studying the awareness of early signs, symptoms and risk factors of oral cancer can really aid in preventing the disease, minimizing the problem consequences and help to establish preventive community program. This study aimed to investigate the awareness of oral cancer regarding knowledge of signs, symptoms and risk factors and source of information, in addition to previous oral cancer screening and attitude towards it among patients attended UST Dental Teaching Hospital during the year 2015. However, to ensure effectiveness of preventive programs; the understanding of the awareness the public of oral cancer risk factors, signs and symptoms is a first step in the process of behavioral change which can lead to avoid them.

## Methods

This hospital-based cross-sectional study was carried out during the year 2015; using an interview questionnaire-based to survey patients attending UST Dental Teaching Hospital at the Faculty of Dentistry, Omdurman.

### Sampling procedure

A total of 500 participants were recruited using a census sampling procedure. Participants were adult dental patients (≥18 years) who agreed to take part and signed the consent. Patients who attended the hospital on emergency basis and those with communication disabilities were excluded from the study. The interviews were conducted during their presence at the hospital.

### Data collection

The data collection was supervised by trained personnel (authors). In order to make each participant feel as comfortable as possible, they were interviewed privately after a brief explanation of the objectives of the study and also responding to their questions and concerns. Interviewer-administered questionnaire was adapted from previously validated items that have been applied in similar studies [20–22]. The questionnaire was adapted to make it suitable for the local population of Omdurman city, especially in the part of risk factors associated with oral cancer. The questionnaire was comprised of close ended questions. It was divided into sections; demographic characteristics, knowledge of oral cancer signs, symptoms, risk factors, oral cancer screening experience and attitude towards the screening and believes about oral cancer (Additional file 1). A pilot study was performed on a sample of dental patients (*n* = 30) attended the UST Dental Teaching Hospital, and the and the relevant and needed amendments, were performed for the final questionnaire.

### Questions and variables

#### Demographic characteristics

*Age group* assessed by the question “*what is your age*” response options recoded into (0) “< 40 years”, (1) “≥ 40 years”.

*Education level* assessed by the question “*what is your education level*” using response options (1) “primary level”; (2) “secondary level”; (3) “university”; (4) “post-graduate”. The original categories recoded into (0) basic education (includes responses 1, 2); (1) “university and post-university education” (includes responses 3, 4).

*Employment* was assessed via the question “*what is your occupation*” using response options (1) “students”; (2) “labor”; (3) “employee”; (4) “unemployed”; (5) “professional”; (6) “retired”. The original categories recoded into (0) unemployed (including original categories 4, 6); (1) employed (including original categories 1, 2, 3, 5).

*Residence* was assessed via the question “*where is your residence*” using response options (1) “suburban”; (2) “urban”; (3) “city”. The original categories recoded into (0) suburban (including the original categories 1); (1) urban (including the original categories 2, 3).

*Sources of information* was assessed via the question; *“from where did you get this information”* using response options (1) “general media (TV, radio)”, (2) “internet (social media)”, (3) “health workers”, (4) “news- paper and magazine”, (5) “other people”. The original categories recoded into (0) from media (includes original response 1, 2, 4); (1) from direct contact (includes 3, 5, 6).

*Heard of oral cancer* was assessed via the question; “*have you heard of oral cancer*”; using response options (1) “yes”; (2) “no”; (3) “I don’t know”. The original categories recoded into (0) no (includes original response 2, 3); (1) yes (includes original response 1)*.*

*Believes about oral cancer* was assessed via *four* questions*;* “*Does the risk factors of oral cancer increase with age*”; “*is oral cancer a preventable disease*”;; using response options (1) “yes”; (2) “no”; (3) “I don’t know”. The original responses recoded into (0) false beliefs (includes original response 2, 3); (1) correct beliefs (includes original response 1); *“is oral cancer is contagious*”; using response options (1) “yes”; (2) “no”; (3) “I don’t know”. The original categories recoded into (0) false beliefs (includes original response 1); (1) correct beliefs (includes original response 2, 3); “*is treatment of oral cancer possible*”; using response options (1) “yes”; (2) “no”; (3) “I don’t know”. The original categories recoded into (0) false beliefs (includes original response 2, 3); (1) correct beliefs (includes original response 1). The sum variable “believes about oral cancer” (Cronbach’s alpha α = .30) was constructed from the questions (0–4), with median split (Median 3, IQR 1), (0) false beliefs (includes original categories 0, 1, 2); (1) correct beliefs (includes original category 3, 4).

*Knowledge of signs and symptoms* was assessed via thirteen questions; “*do you think loss of taste is a sign of oral cancer”; “do you think dry mouth is a sign of oral cancer”; “do you think bleeding from the gum is a sign of oral cancer”; “do you think burning sensation is a sign of oral cancer”; “do you think numbness of the tongue or other area of the mouth is a sign of oral cancer”; “do you think difficulty in chewing or swallowing is a sign of oral cancer”; “do you think an abnormal swelling is a sign of oral cancer”; “do you think soreness in the mouth that bleed easily and doesn’t heal is a sign of oral cancer”; “do you think undue falling or loosing of teeth is a sign of oral cancer”; “do you think continues pain in the jaw is a sign of oral cancer”; “do you think white/red patch on the gum is a sign of oral cancer”; “do you think lump or thickening in the neck is a sign of oral cancer”; “do you think color change is a sign of oral cancer*” using response options (1) “yes”; (2) “no”; (3) “i don’t know”. The original categories recoded into (0) poor knowledge (includes original response 2, 3); (1) good knowledge (includes original response 1). The sum variable “knowledge of signs and symptoms” (Cronbach’s alpha α = .90) was constructed from the 13 questions (0–13) with median split (median7,IQR 6); (0) poor knowledge (includes original categories 0,1,2,3,4,5,6,); (1) good knowledge (includes 7, 8,9,10,11,12,13).

*Knowledge of risk factors of oral cancer* was assessed via eleven questions*; “do you think smokeless tobacco (Toombak) is a risk factor”; “do you think (smoking (cigarette/shisha) is a risk factor”; “do you think alcohol is a risk factor”; “do you think family history of oral cancer is a risk factor”; “do you think exposure to sunlight is a risk factor”; “do you think old age is a risk factor”; “do you think poor oral hygiene is a risk factor”; “do you think chronic trauma is a risk factor”; “do you think sedentary life style is a risk factor”; “do you think hot and spicy food is a risk factor”; “do you think spiritual/demonic attack is a risk factor”*. Using response options (1) “strongly agree”, (2) “agree”, (3) “undecided/neutral”, (4) disagree, (5) “strongly disagree”. The original categories recoded into (0) poor knowledge of risk factors (includes 3, 4, 5); (1) good knowledge of risk factors (includes original response 1, 2). The sum variable “knowledge of risk factor” (Cronbach’s alpha α = .65), was constructed from 11 questions (0–11) with median split (median 5, IQR 2); (0) poor knowledge includes original categories 0, 1, 2, 3, 4); (1) good knowledge (includes original categories 5, 6, 7, 8, 9, 10, 11).

*Attitude towards oral cancer screening* was assessed via the questions; “*do you think oral cancer screening should be mandatory*”; using response options (1) “yes”, (2) “no”, (3) “i don’t know”. The original categories recoded into (0) negative attitude (includes original responses 1, 2); (1) positive attitude (original responses 1).

*Ever screened for oral cancer* was assessed via the question; “*have you ever gone to oral cancer examination (screening)*”; using response options (1) “yes”, (2) “no”, (3) “i don’t know”*.* The original categories recoded into (0) not screened (includes original responses 2, 3); (1) ever screened (original responses 1).

### Data analysis

Data were recorded and analysed using the Statistical Package for Social Science, version 20 (IBM. Chicago, Illinois, USA). Descriptive analyses were performed using frequencies and percentages. For the bivariate analysis chi-square tests were performed to evaluate the categorical variables; the level of significance was set at *p* < 0.05 and 95% confidence intervals (95% CI). Estimates were presented as Odds Ratio (OR) and 95% confidence Interval (CI).

### Ethical consideration

The Ethical Committee of the Faculty of Dentistry, University of Science and Technology, Omdurman, Sudan, approved the study protocol. Written informed consent obtained from all participants. Participation was voluntary, and participants were informed that they could withdraw at any time and that their responses would be anonymous and treated confidentially.

## Results

### Sample profile

As depicted in Table 1; a total of 68% (340) of the participants were < 40-years old, 64.4% (322) were females. More than half of the participants 59% (295) had university level of education. The majority 96.4% (482) was employed and 94.2% (471) were urban residents.

**Table 1:** Percentages and frequencies of demographic characteristics, source of information, oral cancer screening and attitude towards it and knowledge of signs, symptoms and risk factors of oral cancer

Characteristics	Totals % (n)
Age	
< 40	68(340)
≥ 40	32(160)
Gender	
Male	35.6(178)
Female	64.4(322)
Employment	
Not employment	3.6(18)
Employment	86.4(482)
Residence	
Suburban	5.8(29)
Urban	84.2(471)
Source of information	
From media	66.1(290)
From direct contact	33.9(149)
Ever heard of oral cancer	
No	14.4(72)
Yes	85.6(428)
Knowledge of signs and symptoms	
Poor	42.3(210)
Good	57.7(286)
Knowledge of risk factors	
Poor	51(201)
Good	49(193)
Ever screened for oral cancer	
No	93.2(466)
Yes	6.8(34)
Attitude towards oral cancer screening	
Negative attitude	33.6(168)
Positive attitude	66.4(332)
Believes about oral cancer	
False belief	47.5(237)
Correct belief	52.5(262)

Media including TV and internet is the main source of information about oral cancer to those < 40 years (69.8% (215), *p* < 0.05), and those with higher education (69.4% (197), *p* < 0.05).

**Fig. 1:**
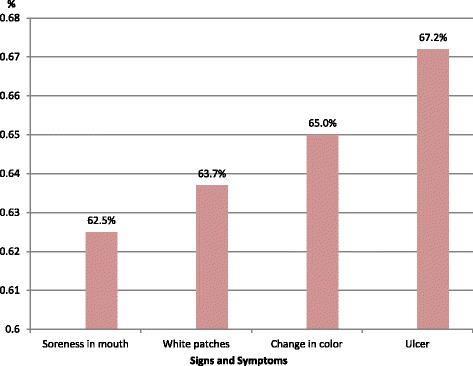
The most common recognized signs and symptoms of oral cancer among the participants

On analysis of individual signs and symptoms of oral cancer as depicted in Table 2, unhealed ulcer reported by 67.2% (336) of the participants, change in color 65% (325), white patches 63.6% (318) and soreness 62.4% (312) were the most common identified signs and symptoms of oral cancer (Fig. 1).

**Table 2:** Percentages and frequencies of recognized signs, symptoms and risk factors of oral cancer

Signs and symptoms	Totals % (n)	Risk factors	Totals % (n)
Dry mouth		Use of Toombak	
No	55.8(279)	No	4.6(23)
Yes	44.2(221)	Yes	95.4(477)
Bleeding		Smoking	
No	45.8(229)	No	10.8(54)
Yes	54.2(271)	Yes	89.2(446)
Burning sensation		Alcohol	
No	58.6(293)	No	20.6(103)
Yes	41.4(207)	Yes	79.4(397)
Numbness		Family history	
No	50.8(254)	No	72.6(363)
Yes	49.2(246)	Yes	27.4(137)
Difficulty in chewing		Exposure to sun	
No	48.9(244)	No	80.6(403)
Yes	51.1(255)	Yes	19.4(97)
Difficulty in swallowing		Aging	
No	51.2(256)	No	69.4(347)
Yes	48.8(244)	Yes	30.6(153)
Soreness in mouth		Bad oral hygiene	
No	37.5(187)	No	29.4(147)
Yes	62.5(312)	Yes	70.6(353)
Teeth loosing		Chronic irritation	
No	46.6(233)	No	71.8(359)
Yes	53.4(267)	Yes	28.2(141)
Pain		Sedentary life	
No	48.9(244)	No	83.6(418)
Yes	51.1(255)	Yes	16.6(82)
White patches		Spicy food	
No	36.3(181)	No	69.8(349)
Yes	63.7(318)	Yes	30.2(151)
Swelling		Spiritual	
No	45(225)	No	78(390)
Yes	55(275)	Yes	22(110)
Change in color			
No	35(175)		
Yes	65(325)		
Ulcer			
No	32.8(164)		
Yes	67.2(336)

Those of < 40 years of age identified ulcer more than their counterparts 70% (238), (OR 0.67, CI0.45–1.00, *p* ≤ 0.05). Identification of change in color as sign and symptom of oral cancer recognized by those with positive attitude towards oral cancer screening scored higher than those with negative attitude 69% (229), (OR 1.66, CI 1.13–2.44, *p* ≤ 0.05); and by females more than males 68.6% (221), (OR 1.55, CI 1.06–2.27, *p* ≤ 0.05), and also those <40 years scored higher than their counterparts 67.9% (231), (OR 0.67, CI 0.45–0.99, *p* ≤ 0.05).

White patches identified by those with positive attitude towards oral cancer screening 69.5% (221), (OR 1.47, CI 1.00–2.15, *p* ≤ 05), those ever heard of oral cancer 89% (283), (OR 2.00, CI 1.21–3.33, *p* ≤ 0.001), and those < 40 years 72% (229), (OR 0.61, CI 0.41–0.90, *p* ≤ 05).

**Fig. 2:**
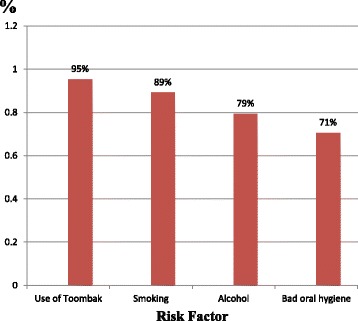
The most common recognized risk factors of oral cancer among the participants

The most common recognized risk factors of oral cancer were illustrated in Fig. 2. When comparing demographic characteristics, the higher education level found to be significantly associated with identifying Toombak as a risk factor for oral cancer as with more than two times likelihood (OR 2.32, CI 0.98–5.48, *p* ≤ 0.05). Also those with positive attitude towards screening reported 67.5% (322), (OR 2.70, CI 1.15–6.29, *p* ≤ 0.05).

Urban residents had better knowledge in identifying smoking as risk factor 90% (446), with almost three times likelihood (OR 2.87, CI 1.16–7.07, *p* ≤ 0.001). Those who heard of oral cancer had better knowledge in identifying smoking as risk factor with likelihood of more than two times than their counterparts (OR 2. 62, CI 1.36–5.06, *p* ≤ 0.001. Females reported alcohol and sedentary life style as risk factors more than males 83.2% (268), (OR 1.88, CI 1.21–2.92, *p* ≤ 0.001) and 18.9% (61), (OR 1.74, CI 1.02–2.98, *p* ≤ 0.05); respectively. Urban residents reported sedentary life style as risk factor more than suburban counterpart 17.2% (81), (OR 5.81, CI 0.78–43.35, *p* ≤ 0.05). Family history reported as risk factor for oral cancer by those whom source of information by direct contact with health workers 34.9% (52), (OR 1.68, CI 1.09–2.59, *p* ≤ 0.05).

Spiritual causes of oral cancer reported by those ≥ 40-years as 30% (48), (OR 1.92, CI 1.24–2.97, *p* ≤ 0.001); and also those with basic education 33.7% (69) (OR 0.31, CI 0.20–0.49, *p* ≤ 0.001).

Demographic characteristics of those reported ever screened for oral cancer; a total of 7.4% (25) of those < 40 years, compared to 5.6% (9) ≥ 40 years; 9% of males compared to 5.6% (18) females; higher educated confirmed screened more than their counterpart; moreover those residents in the urban 7% (33) confirmed screened for oral cancer twice than those suburban residents 3.4% (1), (OR 2.11, CI 0.27–15.99).

Demographic characteristics of those reported positive attitude towards oral cancer screening; 68.9% (222) of females were one and half times likelihood to report positive attitude (OR 1.57, CI 0.93–2.01), those in suburban 82.8% (24) compared to 65.4% (308) in urban. Positive attitude towards oral cancer screening associated with correct beliefs about oral cancer 45.8% (152), (*p* < 0.05). Those confirmed good knowledge of signs and symptoms of oral cancer 62.5% (205) significantly report positive attitude as almost they were near two times likelihood than their counterpart (*p* ≤ 0.001).In addition, those with good knowledge of risk factors 53.9% (137) as one and half likelihood to report positive attitude towards screening of oral cancer Table 3.

**Table 3:** Demographic characteristics, source of information, ever screened, and attitude towards oral cancer screening by good knowledge of signs, symptoms and risk factors of oral cancer

Characteristics	Good knowledge risk factors of oral cancer		Good knowledge of signs and symptoms of oral cancer	
	% (n)	OR (CI)	% (n)	OR (CI)
Age				
< 40	47.0 (124)	1.27 (0.83–1.94)	58.9 (199)	0.85(0.58–1.25)
≥ 40	53.1(69)		55.1 (87)	
Gender				
Male	44.8 (65)		55.7 (98)	1.13(0.78–1.64)
Female	51.4(128)	1.03(0.72–1.47)	58.8(188)	
Employment				
Not employment	35.3 (6)	1.80 (0.65–4.97)	33.3(6)	2.82 (1.04–7.66)
Employment	49.6(187)		58.6 (280)*	
Residence				
Suburban	45.5(10)	1.16 (0.49–2.75)	64.3 (18)	0.74 (0.33–1.64)
Urban	49.2(183)		57.3 (268)	
Source of information				
From media	44.7(101)	1.60 (1.02–2.52)	60.1(173)	0.81(0.54–1.21)
From direct contact	56.5(65)*		55.0% (82)	
Ever heard of oral cancer				
No	48.4(30)	1.02 (0.59–1.77)	54.3(38)	1.17 (0.70–1.95)
Yes	49.1(163)		58.2(248)	
Ever screened for oral cancer				
No	48.9(180)	1.04(0.47–2.31)	57.4 (265)	1.20 (0.58–2.45)
Yes	50(13)		61.8 (21)	
Attitude towards oral cancer screening				
Negative attitude	40 (56)	1.75(1.15–2.66)	48.2 (81)	1.79 (1.22–2.60)
Positive attitude	53.9 (137)**		62.5(205)**	
Believes about oral cancer				
False believe	47.9(90)	1.07(0.72–1.60)	51.2 (122)	1.55 (1.08–2.22)
Correct believe	49.8(102)		62.8 (164)*	

*P ≤ 0.05, **P ≤ 0.001

## Discussion

This study was conducted in UST Dental Teaching Hospital in Omdurman city, Sudan. It is worth noting here that this study is the first of its kind in Sudan. The study provides valuable information about public awareness of risk factors, signs and symptoms of oral cancer.

Among the study participants knowledge of sign and symptoms affected significantly by employment (Table 3), but not affected by other demographic factors which contradicts other studies carried out among Iranians and Malaysians [23–25]. Those younger and females identified ulcers and change in color especially white patches as a sign and symptom for oral cancer better than their counterpart males; that may be contributed to females might be more aware of their well-being, and therefore might be more aware and concerned about any physical changes occurring to their body, in addition to the fact that females are more active in searching for health information than their counterpart male [26, 27].

Awareness of tobacco use (smoked and Toombak) as a risk factor for oral cancer scored high. That may be due to continuous anti-tobacco focus strategies such as ban on advertisements, increase the prices and taxes and restriction areas, which play a role in achieving behavioral change [28].

According to this study almost half of the participants reported negative believes about oral cancer in terms of whether it is contagious, treatable and/or preventable. This is probably due to misinformed public which is a similar situation to what has been revealed in Iran, Yemen and Jordan [23–25, 29].

A considerable portion of the sample in this study received their information via mass media. This in turn indicates the importance of mass media in educating the public and raising awareness about oral cancer signs, symptoms and risk factors, which would entail an increase in early detection, diagnosis and thus survival rates [30]. The results confirm findings published in previous studies reporting that mass media is a common and effective source of information regarding oral cancer [29, 31, 32]. On the other hand, far less individuals obtained information from direct contact with health workers such as dentists. This fact highlights the need for dental professionals to advise and inform their patients about oral cancer. That is consistent with earlier studies carried out on Indian and Italian patients [33, 34].

This study reveals positive attitudes by participants towards screening for oral cancer but it also suggests ignorance of its existence, which is also demonstrated in other studies [35, 36]. Although visual examination proved to be effective in early detection and consequently reduces the mortality rate of oral cancer as a screening program [37], only few individuals of the participants were ever screened in the past for oral cancer. This is similar to has been reported by other studies from different parts of the world [38]. Dentists training and competency to perform oral cancer screening in their routine examinations may be questioned as some studies have emphasized [23, 39, 40]. Using the opportunity provided by a dental appointment to raise awareness may gradually encourage early detection of oral cancer, and as this study has indicated, screening of oral cancer is associated with good knowledge of signs, symptoms and risk factors (Table 1).

This study is not without its limitations, as the findings are based on interview reporting which might be subjected to recall bias and information bias (social desirability). Thus; participants may either have under or over-reported their responses to the questions.

Also, participation of this study was restricted to those who attended to UST Dental Teaching Hospital and this may set some limits on the generalizability of the results, given the nature and composition of the sample. Therefore, the results of this study, although portray an important picture of the current situation, should only be interpreted with caution.

## Conclusion

Awareness levels, knowledge of risk factors and identifying early signs and symptoms of oral cancer necessitate the need for more structured preventive programs using media. Dentists and health workers should do more on this front since they have a pivotal role in early diagnosis by performing oral cancer screening, raising levels of knowledge and in rectifying misconceptions about oral cancer. This would entail a reduction in high rates of morbidity and mortality associated with oral cancer.

## Additional file

Additional file 1: Questionnaire file: contains the questions and consent form in English and Arabic. (DOCX 167 kb)

## Abbreviations

OC: Oral cancer
PMD: Potentially malignant disorders
SLT: Smokeless tobacco
