# Supplementary material for: Oral Cancer Awareness Among Dental Patients in Omdurman, Sudan: a cross-sectional Study
Source: BMC Oral Health. 2017 Mar 23;17:69. doi: 10.1186/s12903-017-0351-z (PMC5364606; doi:10.1186/s12903-017-0351-z)
Supplement: Additional file 1: — Questionnaire file: contains the questions and consent form in English and Arabic. (DOCX 167 kb) [file 12903_2017_351_MOESM1_ESM.docx]

# Additional file 1: Questionnaire

**Introduction:**

This research is aimed to study awareness of signs and symptoms and risk factors of oral cancer and the attitude towards screening among patients attending university of science and technology 2014.

**Consent :**

the purpose of the study was explained to me and confidentiality of the information collected has been assured. I voluntary participated in this study.

Signature ……………………………………………………

***Section A : sociodempgraphic characteristics**

**1-**age………………….

**2-**gender:

female male

**3-**Marital status:

single married divorced widow

**4-**education level:

Primary school secondary school university

Post graduate

**5-**occupation :

Labor employee unemployed professional Retired student

**6-**residence:

Suborn rural urban city

**7-**family/relative/friend history of oral cancer:

Yes no I don’t know

***section B: general awareness**

1**.** Have you heard of oral cancer?

Yes no I don’t know

2**.** Form where did you get this information?

**-**General media (TV, radio) -internet (social media) direct contact -news paper and magazine Other people -others

3**.** is oral cancer a preventable disease?

Yes no I don’t know

1. Is treatment of oral cancer possible?

Yes no I don’t know

6**.** Is oral cancer is contagious?

Yes no I don’t know

6. Does the risk factors of oral cancer increase with age?

Yes no I don’t know

7. Have you ever gone to oral cancer examination (screening)?

Yes no I don’t know

8. Do you think oral cancer screening should be mandatory?

Yes no I don’t know

***section c: signs and symptoms**

1. Do you think Loss of taste is a sign of oral cancer ?

Yes no I don’t know

2. Do you think Dry mouth is a sign of oral cancer ?

Yes no I don’t know

3.Do you think Bleeding from the gum is a sign of oral cancer ?

Yes no I don’t know

4.Do you think Burning sensation is a sign of oral cancer ?

Yes no I don’t know

5.Do you think Numbness of the tongue or other area of the mouth is a sign of oral cancer ?

Yes no I don’t know

6.Do you think Difficulty in chewing or swallowing is a sign of oral cancer ?

Yes no I don’t know

7.Do you think An abnormal swelling is a sign of oral cancer?

Yes no I don’t know

8.Do you think Soreness in the mouth that bleed easily and doesn’t heal is a sign of oral cancer ?

Yes no I don’t know

9.Do you think Undue falling or loosing of teeth is a sign of oral cancer ?

Yes no I don’t know

10. Do you think Continues pain in the jaw is a sign of oral cancer ?

Yes no I don’t know

11. Do you think White /red patch on the gum is a sign of oral cancer ?

Yes no I don’t know

12. Do you think Lump or thickening in the neck is a sign of oral cancer ?

Yes no I don’t know

13. Do you think Color change is a sign of oral cancer ?

Yes no I don’t know

***section D: risk factors and habits**

1. Do you think smokeless tobacco (toombak) is a risk factor?

Strongly agree Agree undecided/neutral disagree strongly disagree

2. Do you think (smoking (cigarette/shisha) is a risk factor?

Strongly agree Agree undecided/neutral disagree strongly disagree

3. Do you think alcohol is a risk factor?

Strongly agree Agree undecided/neutral disagree strongly disagree

4. Do you think family history of oral cancer is a risk factor?

Strongly agree Agree undecided/neutral disagree strongly disagree

5. Exposure to sunlight:

Strongly agree Agree undecided/neutral disagree strongly disagree

6. Do you think old age is a risk factor?

Strongly agree Agree undecided/neutral disagree strongly disagree

7. Do you think poor oral hygiene is a risk factor ?

Strongly agree Agree undecided/neutral disagree strongly disagree

8. Do you think chronic trauma is a risk factor?

Strongly agree Agree undecided/neutral disagree strongly disagree

9. Do you think sedentary life style is a risk factor?

Strongly agree Agree undecided/neutral disagree strongly disagree

10. Do you think hot and spicy food is a risk factor?

Strongly agree Agree undecided/neutral

disagree strongly disagree

11. Do you think spiritual/demonic attack is a risk factor?

Strongly agree Agree undecided/neutral disagree strongly disagree

**مقدمة:**

الهدف من هذا البحث دراسة و قياس الوعي بعلامات وأعراض و العوامل المسببة لسرطان الفم و الموقف العام من الفحص عند المرضى في العيادة التشخيصية في مستشفى الأسنان التعليمي بجامعة العلوم و التقانة.

**الموافقة :**

لقد تم شرح الغرض من هذه الدراسة وتم التأكيد على السرية التامة للمعلومات التي تم جمعها.لقد شاركت في هذا البحث بموافقتي التامة.

التوقيع :..................................................................

**القسم (أ): الصفات الاجتماعية**

1.العمر:..............

2.الجنس:

أنثي ذكر

3.الحالة الاجتماعية:

أعزب متزوج مطلق أرمل

4.المستوي التعليمي:

أساس ثانوي جامعي بعد الجامعي

5.العمل:

عامل موظف محترف متقاعد عاطل عن العمل طالب

6.السكن:

مدن فرعيه أرياف المدينة

7.هل لديك احد من عائلتك ؟أصدقائك ؟معارفك يعاني من مرض سرطان الفم ؟

نعم لا لااعلم

**القسم(ب):معلومات عامة:**

1. هل سمعت عن سرطان الفم؟

نعم لا لااعلم

2.من أين حصلت علي هذه المعلومات؟

-الوسائط العامة(التلفاز / راديو) - وسائط خاصة (انترنت) -الاتصال المباشر -الصحف والمجلات-أشخاص-أخري

3.هل سرطان الفم من الأمراض التي يمكن منع حدوثها؟

نعم لا لا اعلم

4.هل يمكنمعالجة سرطان الفم ؟

نعم لا لا اعلم

5.هل سرطان الفم معدي؟

نعم لا لا اعلم

6.هل خطر حدوث سرطان الفم يزيد بزيادة العمر ؟

نعم لا لا اعلم

7.هل تعتقد أن فحص سرطان الفم ينبغي أن يكون إلزاميا؟

نعم لا لا اعلم

8.هل سبق أن خضعت لاختبار للكشف عن سرطان الفم؟

نعم لا لا اعلم
